# AMP Activated Protein Kinase Is Indispensable for Myocardial Adaptation to Caloric Restriction in Mice

**DOI:** 10.1371/journal.pone.0059682

**Published:** 2013-03-19

**Authors:** Kai Chen, Satoru Kobayashi, Xianmin Xu, Benoit Viollet, Qiangrong Liang

**Affiliations:** 1 Cardiovascular Health Research Center, Sanford Research, University of South Dakota, Sioux Falls, South Dakota, United States of America; 2 Inserm, U1016, Institut Cochin, Paris, France; 3 Cnrs, UMR8104, Paris, France; 4 Univ Paris Descartes, Sorbonne Paris Cité, Paris, France; Temple University, United States of America

## Abstract

Caloric restriction (CR) is a robust dietary intervention known to enhance cardiovascular health. AMP activated protein kinase (AMPK) has been suggested to mediate the cardioprotective effects of CR. However, this hypothesis remains to be tested by using definitive loss-of-function animal models. In the present study, we subjected AMPKα2 knockout (KO) mice and their wild type (WT) littermates to a CR regimen that reduces caloric intake by 20%–40% for 4 weeks. CR decreased body weight, heart weight and serum levels of insulin in both WT and KO mice to the same degree, indicating the effectiveness of the CR protocol. CR activated cardiac AMPK signaling in WT mice, but not in AMPKα2 KO mice. Correspondingly, AMPKα2 KO mice had markedly reduced cardiac function during CR as determined by echocardiography and hemodynamic measurements. The compromised cardiac function was associated with increased markers of oxidative stress, endoplasmic reticulum stress and myocyte apoptosis. Mechanistically, CR down-regulated the expression of ATP5g2, a subunit of mitochondrial ATP synthase, and reduced ATP content in AMPKα2 KO hearts, but not in WT hearts. In addition, CR accelerated cardiac autophagic flux in WT mice, but failed to do so in AMPKα2 KO mice. These results demonstrated that without AMPK, CR triggers adverse effects that can lead to cardiac dysfunction, suggesting that AMPK signaling pathway is indispensible for energy homeostasis and myocardial adaptation to CR, a dietary intervention that normally produces beneficial cardiac effects.

## Introduction

Caloric restriction (CR) is a robust anti-aging intervention. It refers to the practice that reduces calorie intake by 20–50% without causing malnutrition. The ability of CR to extend lifespan was first reported in rats 95 years ago [1], which has since been reproduced in diverse model systems [2]–[4]. Nevertheless, the life extension effect of CR in long-lived primates such as rhesus monkeys remains controversial [5], [6], casting some doubt on the hope that CR may be an effective approach to promote longevity in humans. Despite this uncertainty, however, ample evidence indicates that CR can produce dramatic cardiovascular benefits in humans [7]–[10]. Indeed, CR can lower blood pressure, increase insulin sensitivity, improve blood lipid profile, and suppress atherosclerosis [7], [11], [12]. In addition, CR is sufficient to reduce heart mass and attenuate ventricular chamber stiffness thereby improving diastolic cardiac function in humans [13]–[15]. Animal studies further show that CR is able to not only inhibit age-related cardiac pathology including oxidative injury and hypertrophy [16]–[19], but also reduce acute ischemic injury and chronic cardiac remodeling [20]–[23], demonstrating the ability of CR to protect against age or stress-induced heart disease. Recent research has focused on developing drugs that mimic CR health-promoting effects without reducing food intake [24], [25]. However, the mechanisms of cardioprotection by CR remain speculative, making it hard to design mimetics for harnessing the full benefits of CR.

CR triggers an adaptive response that mobilizes cell defense and repair systems in a coordinated fashion to achieve a state termed hormesis [3], [26], leading to a general attenuation of oxidative damage [27], inflammation [28], and apoptosis [29], which is associated with reduced serum levels of several hormones including insulin and insulin-like growth factor 1 (IGF-1) [3], [30]. CR-induced hormesis must obviate the potential negative effects of CR and redirect molecular priorities to pathways designed for energy saving, stress resistance, repair, and survival [31]. Accordingly, a defect in CR signaling cascade could potentially undermine the ability of CR to achieve the hormetic state and protect the heart.

AMPK is a heterotrimeric protein kinase composed of a catalytic α subunit and two regulatory subunits (β & γ). As an energy sensor, AMPK detects and reacts to fluctuations in intracellular ATP levels under normal and stress conditions. CR-induced relative energy deficits result in elevated intracellular AMP and ADP, which bind to the γ subunit, leading to phosphorylation of the α subunit on its activating loop at Thr-172 by a upstream kinase such as LKB1, thereby activating AMPK. The activated AMPK affects multiple metabolic pathways to maintain an energy homeostasis conducive to stress resistance and cell survival [32]. Not surprisingly, AMPK has been suggested to play a role in CR-induced longevity [7], [33]–[36] and myocardial protection [19], [21] based on the correlation between the activated AMPK and the observed effects. However, definitive evidence is lacking to support the hypothesis that the cardioprotective effect of CR under disease conditions is mediated through AMPK pathway. It is also unclear if AMPK is really important for mounting an effective hormetic response to maintain cardiac homeostasis during periods of restricted calorie intake under basal physiological conditions. Therefore, in the present study we tested the necessity of AMPK in myocardial adaptation to CR by using AMPK deficient mice. Our results demonstrated that AMPK activation is absolutely required for sustaining energy production during CR to maintain a normal cardiac function.

## Materials and Methods

### Reagents and Antibodies

Bafilomycin A1 (BFA) was obtained from LC laboratories (Woburn, MA). Anti-nitrotyrosine and anti-4-hydroxynonenal antibodies were from EMD Millipore (Billerica, MA). Antibodies against ATP synthase Fo complex subunit C2 (Atp5g2), cytochrome c oxidase subunit IV (COX-IV), Cytochrome b5 (Cytb5), and optic atrophy type 1 (OPA1) were purchased from Abcam (Cambridge, MA). Anti-dynamin-related protein 1 (Drp1) antibody and horseradish peroxidase (HRP)-conjugated secondary antibodies anti-rabbit, anti-mouse, and anti-goat IgG were obtained from Santa Cruz Biotechnology (Santa Cruz, CA). Anti-p62 c-terminus/SQSTM1 antibody was purchased from Research Diagnostics (Flanders, NJ). The following antibodies were purchased from Cell Signaling (Danvers, MA): AMPKα, AMPKα1, AMPKα2, Phospho-AMPKα (Thr172), AMPKβ1/2, Phospho- AMPKβ1 (Ser108), Acetyl-CoA Carboxylase (ACC), Phospho-ACC (Ser79), Tuberin/Tuberous sclerosis protein 2 (TSC2), Phospho-TSC2 (Ser1387), microtubule-associated protein light chain 3 (LC3), C/EBP homologous protein (CHOP), 78 kD glucose-regulated protein (GRP78) or BIP, Protein disulfide isomerase (PDI), RNA activated protein kinase (PKR)-like endoplasmic reticulum kinase (PERK), Inositol-Requiring Enzyme 1 Alpha (IRE1α), Unc-51-like kinase 1 (ULK1), Phospho-ULK1 (Ser555), Beclin 1, Atg 5, Atg7, Atg12, Atg16L1, and Glyceraldehyde-3-phosphate dehydrogenase (GAPDH).

### Animals and Caloric Restriction Protocols

AMPKα2 knockout (KO) mice and their wide type (WT) littermates were used in the study [37]. The AMPKα2 KO mice have been backcrossed to C57B/6J strain for more than 8 generations. Twelve-week-old mice were fed ad libitum (AL) with the #2018 rodent diet for 1 week. The average daily food intake was used as the base to calculate the amount of food to be allocated to mice that would undergo caloric restriction (CR). Mice were randomly assigned to AL and CR groups. AL mice continued to be fed ad libitum. CR mice were fed 20% less than consumed by AL groups for 2 weeks, followed by 40% less for another 2 weeks. Animals in CR groups were given food at 5∶00 p.m. every day. The allocated amount of food was updated weekly based on food consumption in AL group. At the end of the study, cardiac function was measured with echocardiography and hemodynamics. Mice were sacrificed and blood samples were drawn. The hearts were quickly removed, washed, weighed and cut into pieces for subsequent analyses.

### Ethics Statement

All procedures with animals were performed according to the Public Health Service Guide for Care and Use of Laboratory Animals and were approved by Sanford Research/USD Institutional Animal Care and Use Committee.

### Measurement of Cardiac Function

Echocardiography and hemodynamics were performed as described previously [38], [39]. in brief, echocardiographic measurements were performed under anesthesia (3% isoflurane induction, 1% maintenance) using a visualsonics vevo 2100 high-resolution imaging system (visual sonics, toronto, canada). Left ventricular dimensions and heart rate were measured from 2-d short-axis m-mode tracings at the level of the papillary muscle. Left ventricular mass and functional parameters were calculated using the above primary measurements. Left ventricular hemodynamics were measured under anesthesia using a millar catheter (millar instruments, houston, tx) inserted into the left ventricle via the carotid artery. After stabilization, heart rate (hr) as well as left ventricular end-systolic and end-diastolic pressure were measured from the left ventricular pressure waveforms, and ±dp/dt were calculated.

### Western Blot Analysis

Protein samples from the left ventricle were prepared in lysis buffer as described previously [40]. The proteins (40–50 µg) were resolved by SDS-PAGE and transferred to a PVDF membrane (GE Healthcare). The membrane was then incubated with a primary antibody and followed by a HRP-conjugated secondary antibody. Specific proteins were detected by chemiluminescent methods using the Lumigen TMA-6 kit (Lumigen, Southfield, Michigan). Protein abundance on western blots was quantified by densitometry using Quantity One software (Bio-Rad, Hercules, CA).

### TUNEL Assay and DNA Laddering Assay

Apoptotic cells were detected by terminal deoxynucleotidyl transferase-mediated nick-end labeling (TUNEL) using the APO-BrdU TUNEL Assay Kit (Molecular Probes Inc, Eugene, OR) as per the manufacturer’s protocol. Briefly, Hearts were cut into pieces and embedded in OCT media (Sakura Finetechnical Co., Ltd. Japan). Frozen ventricular sections (5 µm) were fixed in 4% (w/v) paraformaldehyde for 15 min on ice, permeabilized with 70% ethanol for 30 min on ice, and incubated with 50 µL DNA-labeling solution containing TdT enzyme and Br-dUTP at 37°C for 60 min. After the labeling reaction, the sections were washed and stained with fluorescein-labeled anti-BrdU antibody for 30 min. Before mounting, the cells were stained with 4′, 6-diamidino-2-phenylindole (DAPI) and Alexa Fluor 594-labeled phalloidin. Images were captured at 600X using an FV1000 confocal microscope (Olympus, Japan) and percentage of TUNEL positive cells were measured using Image J (NIH) from 4–5 regions per heart, an area of at least 100 cardiac myocytes. Apoptosis-induced DNA fragmentation in the heart was also assessed by using a semiquantitative PCR-based DNA laddering kit from Maxim Biotech, Inc (San Francisco, CA) as described previously [41].

### Markers of Oxidative Stress

Protein carbonyl content in the heart homogenates was measured by western blot analysis using the Oxyblot™ Protein Oxidation Detection Kit (S7150, Chemicon, Temecula, CA), based on immunochemical detection of protein carbonyl groups derived from 2,4-dinitrophenyl hydrazine [42]. Lipid peroxidation and protein nitration were evaluated by standard Western blotting procedure using 4-Hydroxynonenal (4-HNE) and 3-Nitrotyrosine (3-NT) antibodies respectively.

### Measurement of Serum Levels of Insulin, IGF-1 and Triglyceride

Serum levels of insulin, IGF-1 and triglyceride were determined by using commercial kits following the manufacturer’s protocols (rat/mouse insulin ELISA KIT: Millipore, St. Charles, MO; mouse/rat IGF-1 ELISA kit: Diagnostic Systems Laboratories, Webster, TX; and triglycerides liquid reagent set: Pointe Scientific, Canton, MI). The absorbances were measured at 450 nm for insulin and IGF-1, 500 nm for triglyceride on a Synergy™ Mx multi-mode microplate reader from BioTek. The concentrations were calculated based on standard curves generated at the same time.

### Measurement of Tissue ATP

The tissue levels of ATP were measured by using a colorimetric/fluorometric assay kit (Abcam, San Francisco, CA). Briefly, the left ventricle tissues were lysed with ATP assay buffer and centrifuged at 15,000xg for 2 minutes to pellet insoluble materials. The supernatant and 50 µl of the reaction mix were added to each well on the 96-well plate. The absorbance was read at 535/587 nm using a Synergy™ Mx multi-mode microplate reader from BioTek. The ATP contents were calculated based on a standard curve generated at the same time.

### Statistical Analysis

Quantitative data were presented as the Means ± SE. Differences between experimental groups were examined by two-way analysis of variance (ANOVA) followed by the Bonferroni post-test using Prism software (GraphPad). For all analysis, p<0.05 were considered statistically significant.

## Results

### Four Weeks of CR Reduced Mouse Body Weight and Serum Levels of Insulin and IGF-1

Long-term CR in animals induces a host of changes at organ, cellular and molecular levels, among which are reduced body weight (BW) and serum levels of several hormones including insulin and IGF-1 [3], [30]. We determined if a short-term CR could produce similar effects on mice. Three-month old male C57BL/6J mice were fed #2018 rodent diet (Harlan) either ad libitum (AL) or at a restricted amount (CR) for 4 weeks, i.e., 20% less food intake than AL for 2 weeks followed by 40% less for 2 weeks. This short-term CR reduced serum levels of insulin and IGF-1 by 79% and 39%, respectively (Table 1), validating the effectiveness of the CR protocol. In addition, CR caused a roughly 20% decrease in both BW and heart weight (HW) without an effect on tibial length (TL). Consequently, the HW/BW ratio remained unchanged but the HW/TL ratio was reduced after CR (Table 2). Despite a smaller heart, the cardiac function was well maintained during CR as indicated by the normal fractional shortening (FS) measured with echocardiography (Table 2). In summary, the 4-week CR regimen was sufficient to produce hallmark features of long-term CR in mice.

**Table 1:** Effects of CR on serum levels of insulin and IGF-1.

Parameter	AL	CR
N (mice)	6	6
Insulin (ng/ml)	0.59±0.03	0.12+0.03**
IGF-1 (ng/ml)	476.9±22.3	294.8±21.1**

** p<0.01 vs AL group.

**Table 2:** Cardiac gravimetric and echocardiographic data.

Parameter	AL	CR
N (mice)	12	12
HW (mg)	120±4.2	93.3±1.6**
BW (g)	26.3±0.6	20.4±0.3**
TL (mm)	22.2±0.01	22.1±0.01
HW/BW	4.56±0.11	4.57±0.07
HW/TL	5.39±0.18	4.23±0.06**
HR (bpm)	470±7	457±6
LVIDd (mm)	3.75±0.07	3.41+0.07**
LVIDs (mm)	2.39±0.06	2.10±0.06**
FS(%)	36.3±0.8	38.4±0.6

HW indicates heart weight; BW, body weight; TL, tibial length; HR, heart rate; LVIDd, left ventricular (LV) internal diameter in diastole;; LVIDs, LV internal diameter in systole; FS, fractional shortening. Data are presented as Mean±SE and analysized by t test.

* p<0.05,

** p<0.01 vs AL group.

### Four Weeks of CR Activated AMPK Signaling Pathway in the Mouse Heart

**Figure 1:**
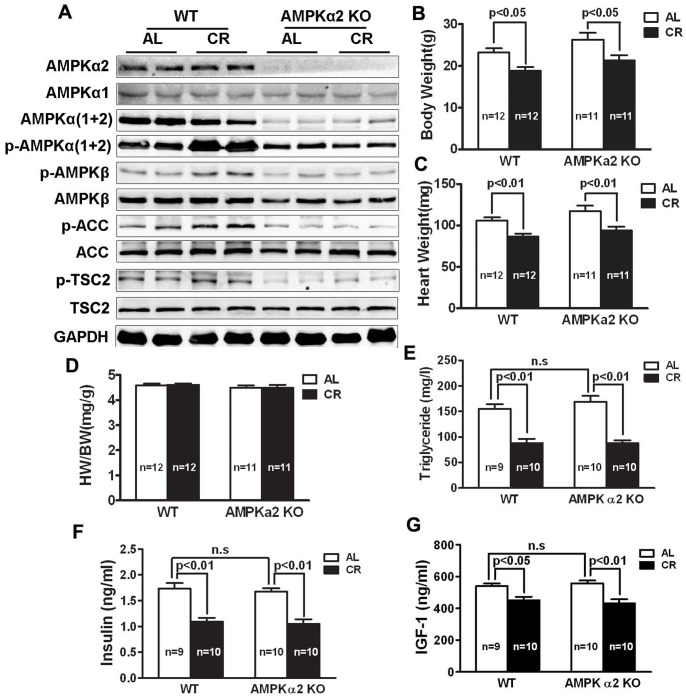
General effects of CR on AMPKα2 KO mice and their WT littermates. Both AMPK α2 KO mice and C57B/6 WT littermates were either fed ad libitum (AL) or subjected to CR (mice were allocated 20% less food than AL for 2 weeks and 40% less for another 2 weeks). Western blot analysis of cardiac proteins was performed to assess the total protein levels and the phosphorylation states of AMPK α1/2 and β subunits as well as its downstream target acetyl-CoA carboxylase (ACC) and TSC2 (**A**). Note: the antibodies against AMPKα and p-AMPKα can recognize both α1 and α2 isoforms. Other parameters indicative of the effectiveness of the CR protocol include body weight (BW, **B**), heart weight (HW, **C**), HW/BW ratio (**D**), blood triglycerides (**E**), and serum levels of insulin (**F**) and IGF-1 (**G**). Data areexpressed as mean ± SE and analyzed by two-way ANOVA. Sample sizes were indicated in the bar graphs.

Different CR protocols have been shown to activate cardiac AMPK [19], [21], [43], an energy sensor essential for maintaining energy homeostasis. Consistently, the 4-week CR regimen in the present study was sufficient to activate AMPK in the mouse heart as indicated by the increased phosphorylation of α and β subunits and its downstream target acetyl-CoA carboxylase (ACC) and TSC2, suggesting that AMPK may play a role in maintaining cardiac homeostasis during CR (left half of the Western blot in Fig. 1A).

### AMPK Activation is Required to Maintain Normal Cardiac Function in Response to CR

Upon activation by CR-induced low energy status, AMPK switches on ATP producing catabolic pathways such as fatty acid oxidation and glucose transport, and at the same time shuts off anabolic ATP consuming pathways such as lipogenesis and protein synthesis [32]. By doing so, AMPK may offset the initial energy deficit induced by CR thereby helping keep a normal cardiac function during extended CR. However, the necessity of AMPK activation for maintaining cardiac homeostasis during CR has never been directly determined. Therefore, we used AMPKα2 knockout (KO) mice [32] to investigate the role of AMPK in the heart during CR. AMPK has two isoforms of the catalytic α-subunit (α1 and α2). AMPKα2 KO mice show no detectable α2 protein and have markedly reduced cardiac protein levels of total and phosphorylated AMPKα (α1+α2), indicating α2 as the dominant isoform in the heart (Fig. 1A). Deletion of AMPK α2 leads to decreased phosphorylation of ACC and TSC2, confirming the diminished AMPK signaling in the α2 KO heart. These mice do not have an appreciable baseline cardiac phenotype although they are sensitive to pressure overload-induced cardiac remodeling [44].

Wild type (WT) and AMPKα2 KO mice were fed either ad libitum (AL) or subjected to CR as described earlier. CR induced AMPK activation in the hearts of WT mice but not AMPKα2 KO mice (Fig. 1A). In response to CR, both WT and KO mice had similarly reduced BW (Fig. 1B), HW (Fig. 1C), blood triglycerides (Fig. 1E), and serum levels of insulin (Fig. 1F) and IGF-1 (Fig. 1G), indicating the effectiveness of the CR protocol. We assessed and compared cardiac function between WT and KO mice with or without CR by echocardiographic and hemodynamic analyses. As shown in Table 3, both cardiac systolic and diastolic function were markedly reduced in AMPKα2 KO mice as compared to WT in response to CR. The impaired cardiac function in the KO mice was indicated by a 26% reduction in fractional shortening (FS) and ejection fraction (EF), a 10% and 24% decrease in left ventricular systolic pressure (LVSP) and +dP/dt, respectively, a 30% reduction in –dP/dt, a 39% increase in LV end diastolic pressure (LVEDP), and a 68% increase in Tau. Together, these results suggest that AMPK activation is required to maintain normal cardiac systolic and diastolic function during CR.

**Table 3:** Cardiac echocardiographic and hemodynamics data in WT and AMPKα2 KO mice with or without caloric restriction.

Parameter	WT-AL	WT-CR	KO-AL	KO-CR
Heart Rate (bpm)	490±12	427±15**	488±10	426±14# #
LVESD (mm)	2.00±0.14	1.81±0.10	2.04±0.14	2.58±0.10# # $$
LVEDD (mm)	3.31±0.17	3.13±0.12	3.37±0.17	3.67±0.12$$
LVESV(µl)	13.8±2.0	10.5±1.6	14.4±2.0	24.7±2.5# # $$
LVEDV (µl)	46.4±5.5	39.9±4.0	48.1±5.6	57.9±4.7$$
EF (%)	71.7±2.0	74.6±1.7	77.4±1.8	57.8±1.3# # $$
FS (%)	40.2±1.7	42.4±1.4	39.9±1.6	29.9±0.8# # $$
Stroke Volume (µl)	32.6±3.5	29.4±2.6	33.7±3.6	33.2±2.4
Cardiac Output(ml/min)	16.1±1.9	12.6±0.9	16.2±2.0	14.2±0.9
N (mice)	11	11	10	10
+dp/dt max	11573±178	10895±398	10892±439	8271±474# # $$
-dp/dt min	10263±344	9591+335	10522±331	7286±593# # $$
Tau (ms)	7.4±0.3	8.2±0.3	8.6±0.4	14.1±2.4# $
LVSP	112.6±1.8	109.8±2.0	117.6±1.8	101.9±2.4# # $
LVEDP	9.0±1.0	8.6±0.8	8.6±1.3	12.1±1.0# $
N (mice)	9	9	9	9

LVESD indicates left ventricle (LV) end systolic diameter; LVEDD, LV end diastolic diameter; LVESV, LV end systolic volume; LVEDV, LV end diastolic volume; EF, ejection fraction; FS, fractional shortening; LVSP, LV systolic pressure; LVEDP, LV end diastolic pressure. Data are presented as Mean±SEM and analysized by 2-way ANAVO with the Bonferroni post-tests.

* p<0.05,

** p<0.01 vs WT-AL;

# p<0.05,

## p<0.01 vs AMPKα2 KO-AL;

$ p<0.05,

$$ p<0.01 vs WT-CR.

### AMPK Activation is Essential for Maintaining Cardiac ATP Homeostasis during CR

**Figure 2:**
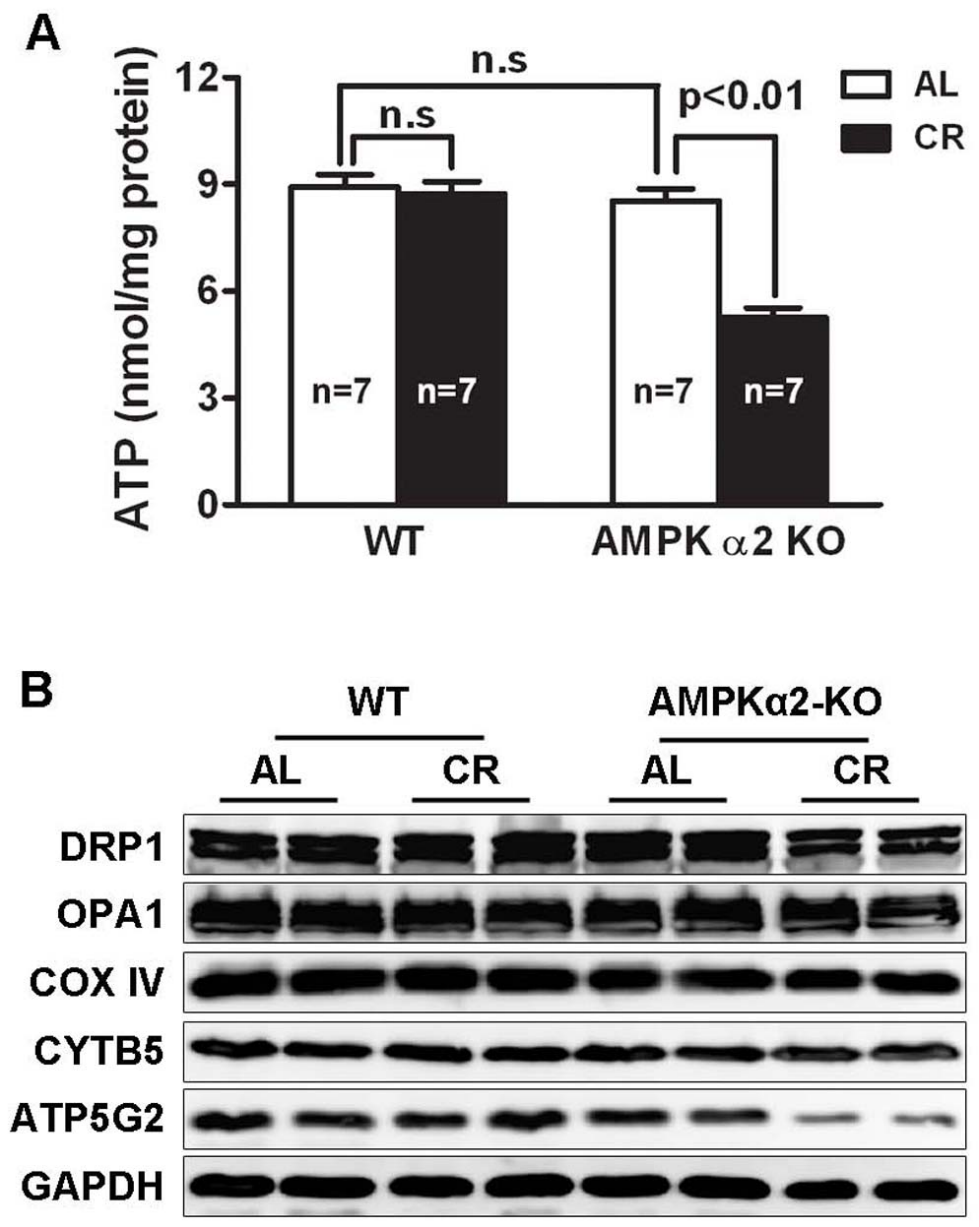
Cardiac ATP contents and Atp5g2 expression levels were reduced in AMPKα2 KO mice during CR. **A.** Cardiac ATP contents were determined with a colorimetric/fluorometric assay kit (Abcam, San Francisco, CA). Data are expressed as mean ± SE and analyzed by two-way ANOVA. **B.** Western blot analysis of proteins involved in regulating mitochondrial function and dynamics.

Since AMPK is the key player that senses energy deficit and controls ATP homeostasis, we determined if the impaired cardiac function in AMPKα2 KO mice during CR was linked to reduced ATP content. As shown in Figure 2A, despite no difference in WT mice between the two feeding groups, the cardiac ATP levels were decreased by 38.5% in AMPKα2 KO mice subjected to CR as compared with the mice fed ad libitum (AL). Mitochondria are the major site for ATP generation. To explore the potential defects that may underlie the reduced ATP levels in AMPKα2 KO heart, we screened proteins involved in several mitochondrial processes such as fission (Drp1), fusion (Opa1), and oxidative phosphorylation (Cytochrome c oxidase IV: Cox IV; Cytochrome b5: Cytb5; ATP synthase Fo complex subunit C2: Atp5g2). As determined by Western blot analysis, CR led to down-regulated protein expression of Atp5g2 in AMPKα2 KO mice, but not in WT mice (Fig. 2B). Together, these results suggest that AMPK is essential for the animal to maintain cardiac ATP homeostasis during CR, in part due to the effects of AMPK on mitochondrial ATP synthase.

### Diminished AMPK Signaling Increased Cardiac Oxidative Stress, ER stress and Apoptosis during CR

**Figure 3:**
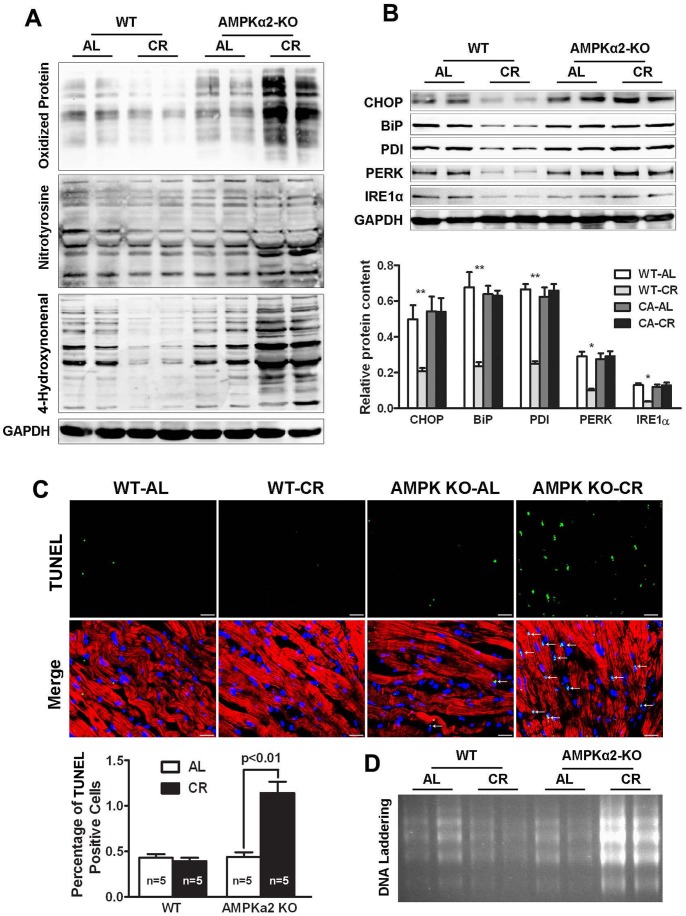
Knocking out AMPKα2 increased cardiac oxidative stress, ER stress and apoptosis during CR. Both AMPKα2 KO and WT mice were subjected to AL or CR feeding as described in Figure 1. **A.** Western blot analysis of protein carbonyl groups (upper), nitrotyrosine (middle) and 4-hydroxynonenal (bottom). **B.** Western blot analysis of proteins involved in ER stress including CHOP, BiP, PDI, PERK, and IRE1α. Data are expressed as mean ± SE (*n* = 4) and analyzed by two-way ANOVA, *p<0.05; **p<0.01 vs WT-AL group. **C.** TUNEL labeling. Apoptotic nuclei were stained green, and slides were counter-stained with Alexa fluor 568 conjugated-phalloidin (red) and DAPI (blue). Scale bars are 20 µm. TUNEL positive cells were expressed as mean ± S.E and analyzed by 2-way ANOVA (*n* = 5). **D.** DNA laddering. A semiquantitative PCR-based DNA laddering kit was used to assess the degree of apoptosis in the heart.

CR-induced hormetic response has been shown to attenuate oxidative stress and ER stress in several organs including the heart [45]. We determined if this beneficial effect of CR could be affected by the loss of AMPK signaling. We assessed cardiac protein oxidation by measuring carbonyl groups, a hallmark of reactive oxygen species modified proteins, nitrotyrosin (NT), a product of tyrosine nitration mediated by reactive nitrogen species such as peroxynitrite anion and nitrogen dioxide, and 4-Hydroxynoneal (4-HNE), a product of lipid peroxidation. As shown in Figure 3A, CR reduced the formation of protein carbonyls and 4-HNE in WT mice, but this effect was abolished in AMPKα2 KO mice. In fact, the levels of protein carbonyl groups, NT and 4-HNE in AMPKα2 KO hearts were all increased when animals were subjected to CR, suggesting that the diminished AMPK signaling undermined the ability of CR to inhibit oxidative stress. Similarly, we investigated the effect of CR on cardiac ER stress in both WT and AMPK α2 KO mice. As shown in Figure 3B, CR reduced the expression levels of ER stress markers in WT hearts including GRP78 or BiP, an ER-specific chaperone, CHOP, a transcriptional factor involved in ER stress response, as well as the 3 upstream components of ER stress, PDI, PERK and IRE1α [46]. However, CR failed to attenuate these ER stress markers in AMPKα2 KO mice. Instead, the ER stress was enhanced in AMPKα2 KO mice during CR. Consistent with the increased oxidative stress and ER stress, cardiac apoptosis was dramatically increased in AMPKα2 KO mice during CR as shown by increased TUNEL labeling (Fig. 3C) and DNA laddering (Fig. 3D). Collectively, these findings indicate that AMPK signaling is required for CR to attenuate oxidative stress, ER stress and apoptosis in the heart.

### AMPK is Essential for CR-induced Autophagy

**Figure 4:**
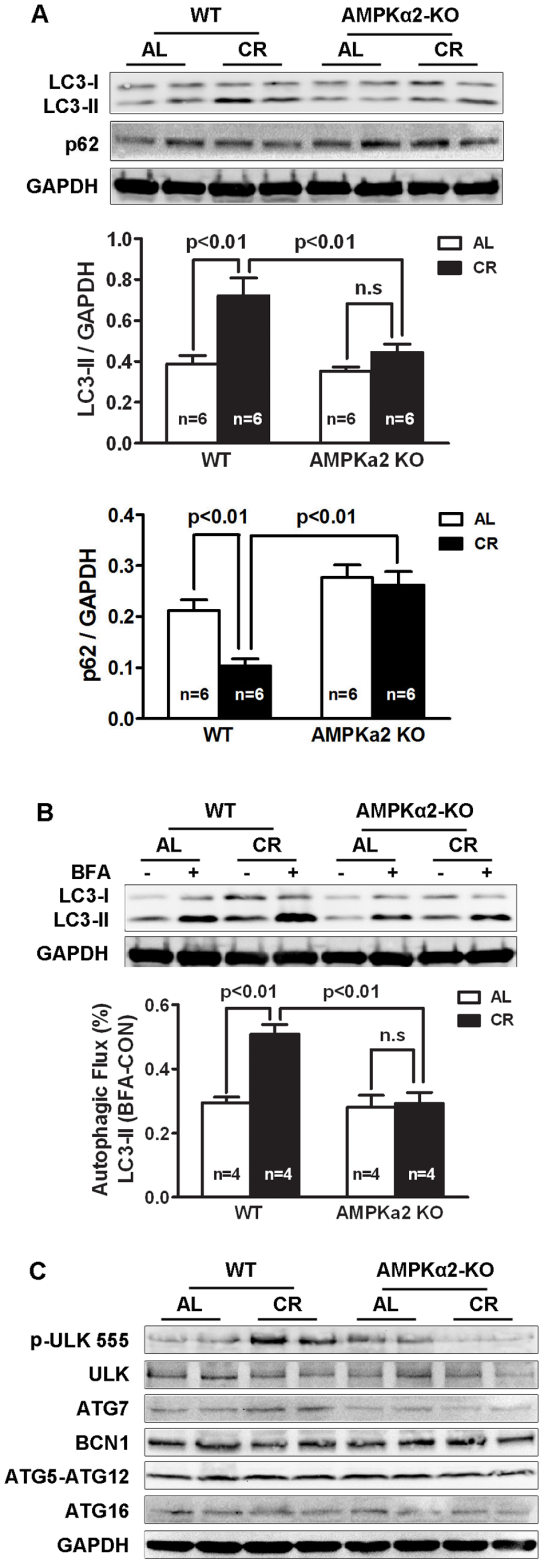
AMPKα2 is essential for the CR-induced autophagy in the heart. **A.** CR increased LC3-II levels and decreased p62 levels in WT mice, but not in AMPKα2 KO mice. **B.** CR increased cardiac autophagic flux in WT mice, but not in AMPKα2 KO mice, as shown by the difference of LC3-II protein levels in the absence and presence of bafilomycin A1 (BFA, 3 µmole/kg), an inhibitor of autophagic degradation. BFA was delivered by i.p. injection 30 minutes before animals were sacrificed. **C.** CR induced the phosphorylation of ULK1 at ser555 in WT mice, but not in AMPKα2 KO mice. Data in A and B were expressed as mean ± SE and analyzed by two-way ANOVA. Sample sizes were indicated in the bar graphs.

Autophagy is the primary cellular pathway for lysosomal degradation and recycling of long lived proteins and organelles. Autophagy is induced to offset energy deficit thereby promoting myocardial survival in response to CR [47], starvation [48] or ischemia [49]. Strong evidence suggests AMPK as a positive regulator of autophagy [49]–[51]. We tested if CR could induce cardiac autophagy in AMPK deficient mice by measuring the protein levels of LC3 and p62. When autophagy is induced, LC3-I is converted to LC3-II and conjugated to the autophagosome membrane. The amount of LC3-II is proportional to the number of autophagic vacuoles that exist in the cell. LC3-II remains associated with autophagic vacuoles until being recycled or degraded by lysosomal proteases. p62/SQSTM1 is a polyubiquitin-binding protein that is degraded by autophagy. Thus, the protein levels of p62 are inversely related to autophagic activity[52]–[55]. As suspected, CR increased cardiac LC3-II levels and decreased p62 levels in WT mice, but these effects disappeared in AMPKα2 KO mice (Fig. 4A),suggesting that autophagy induction in WT mice was impaired by diminished AMPK signaling in KO mice. To ascertain that the changes in LC3-II levels were caused by altered formation of autophagosomes rather than the clearance of autophagic vacuoles, we determined autophagic flux, an index that reflects the number of autophagic vacuoles delivered to and degraded in the lysosome [56]. This was assessed by the difference of LC3-II protein levels in the absence and presence of bafilomycin A1 (BFA, 3 µmole/kg), an inhibitor of autophagic degradation. BFA was delivered by i.p. injection 30 minutes before animals were sacrificed. As shown in Figure 4B. CR-induced increase in LC3-II was further elevated by BFA in WT mice (AL 0.29±0.02 versus CR 0.51±0.03, n = 4, p<0.01); but this effect was markedly diminished in AMPKα2 KO mice (AL 0.28±0.04 versus CR 0.29±0.04, n = 4, p>0.05). These results suggest that the ability of CR to induce autophagy is mediated, at least in part, by AMPK activation.

Unc-51-like kinase 1 (ULK1) is a mammalian homolog of yeast autophagy-related 1 (Atg1) that forms a complex with at least 3 other proteins (Atg13, FIP200 and Atg101) to initiate autophagosome formation [57]–[59]. Phosphorylation of ULK1 by AMPK at Ser-555 appears to be critical for starvation-induced autophagy in cell culture [60], [61]. We examined the phosphorylation state of ULK1 in the mouse heart. As shown in Figure 4C, CR induced the phosphorylation of ULK1 at Ser-555 in WT mice, but not in AMPKα2 KO mice, consistent with the functional status of autophagy in the heart. Of note, the expression levels of several autophagy-related genes, including Beclin-1, Atg5, Atg7, Atg12 and Atg16, were not markedly altered in all four groups. These results suggested a crucial role for AMPK-ULK1 axis in regulating cardiac autophagy under caloric restricted conditions.

## Discussion

Excessive calorie intake posts an increased risk for cardiovascular disease. On the contrary, caloric restriction (CR) can enhance cardiovascular health [2]–[4]. Indeed, CR not only reduces several risk factors for heart disease [7]–[10], but also directly affects cardiac growth and function [13]–[15]. These observations demonstrate a preventative and therapeutic potential of CR for heart disease. However, it has proven difficult to implement and sustain a CR regimen in humans. Thus, research interest has focused on identifying agents that can mimic CR health-promoting effects without reducing calorie intake [24], [25]. In order to design mimetics for harnessing the full benefits of CR, it is imperative to identity the underlying mechanisms that mediate CR-induced cardioprotection.

CR-triggered adaptive response uses diverse signaling mechanisms to achieve a hormetic state that provides cardioprotection at baseline and in response to various stresses [3], [26]. The AMPK signaling pathway has been implicated in CR-induced lifespan extension effects in a number of organisms [7], [33]–[36]. AMPK has also been shown to protect the mouse heart under ischemic or hypertrophic [44], [49], [62], [63] conditions. These studies suggest that the ability of CR to protect the heart may be mediated through AMPK. In support of this hypothesis, the ability of CR to reduce cardiac hypertrophy [19] and ischemic injury [21], [43] was associated with AMPK activation. In addition, either short-term or life-long CR protected the mouse heart against ischemia-reperfusion damage in the Langendorff model, and the cardioprotection was abolished by the AMPK inhibitor adenine 9-β-D-arabinofuranoside (AraA) that was given intravenously [21] or added to the perfusate before the onset of ischemia [43]. Nevertheless, AraA may not be highly specific for AMPK. Also, the necessity of AMPK for CR to mount an effective hormetic response remains largely untested. In this respect, using a genetic loss-of-function approach, we showed that CR activated cardiac AMPK in WT mice but not in mice deficient in AMPKα2; and that WT mice maintained normal cardiac function during CR, but AMPKα2 KO mice lost this capacity. Thus, the diminished AMPK signaling not only undermined the CR-induced hormetic response, but also triggered adverse effects that led to cardiac dysfunction, suggesting an indispensible role for AMPK in myocardial adaptation to CR, a dietary intervention that normally produces beneficial cardiac effects.

The mechanisms by which AMPK provides cardioprotection in the context of CR are not entirely clear. Upon activation by CR, AMPK switches on ATP producing catabolic pathways and concurrently shuts off anabolic ATP consuming pathways, thus keeping ATP at relatively normal levels to maintain cardiac function during CR. Indeed, ATP levels were reduced in AMPKα2 KO hearts, which may have contributed to the impaired cardiac function during CR. This notion is supported by a previous study showing an essential role of ATP metabolism in contractile function and cardiomyocyte survival upon pressure overload [64]. Mitochondrial ATP synthase catalyzes ATP synthesis utilizing an electrochemical gradient of protons across the inner membrane. ATP5g2, a subunit of mitochondrial ATP synthase, was down regulated in AMPKα2 KO mice during CR, which may partially account for the reduced ATP levels. Together, our results demonstrated that AMPK is required for maintaining energy homeostasis, which is essential for preventing cardiac dysfunction in response to restricted food availability.

The compromised cardiac function in AMPKα2 KO mice during CR was also associated with increased markers of oxidative stress, ER stress and myocyte apoptosis, suggesting an essential role for AMPK in mediating CR-induced attenuation of oxidative damage [27] and apoptosis [29]. The protective effects of AMPK are likely mediated through multiple mechanisms, such as stimulation of FoxO, Nrf2, and SIRT1 signaling pathways to improve cellular stress resistance, inhibition of NF-κB signaling to suppress inflammatory responses, and maintenance of mitochondrial quality to reduce free radical generation [65]. Although mitochondrion and oxidative stress are critical for inducing apoptosis in the heart under various conditions, increasing evidence also suggests other organelles as major points of integration of proapoptotic signaling [66], [67]. In particular, endoplasmic reticulum (ER) stress has been shown to play a significant role in the process of apoptosis, contributing to the pathogenesis of heart disease. AMPK is able to attenuate hypoxia-induced ER stress in neonatal rat cardiomyocytes through inactivation of eEF2 [68] and reduce apoptosis during cardiac dysfunction [69], [70]. It is thus not surprising that inactivation of the AMPKα2 gene could turn CR, a normally protective intervention, into a damaging stimulus that triggers oxidative stress, ER stress and apoptosis, leading to cardiac dysfunction as shown in our present study.

Another potential mechanism that could mediate the protective effect of AMPK during CR is the induction of autophagy, the primary cellular pathway for lysosomal degradation and recycling of long-lived proteins and organelles. Autophagy plays an extremely important role in maintaining cell and organ homeostasis under both basal and various stressful conditions [71], [72]. Accumulating evidence suggests that CR may induce autophagy as a means to promote survival and longevity [73], [74]. Supporting this notion, autophagy is required for the survival of starved neonatal mice [48] and for CR-mediated lifespan extension in C. elegans [75]. These results suggest that autophagy may play a role in CR-mediated myocardial protection. Indeed, autophagy is induced to offset energy deficit, promoting myocardial survival in response to CR [47], starvation [48] or ischemia [49]. Autophagy is controlled by multiple signaling pathways including AMPK, a positive regulator, in a number of organisms [49]–[51]. Our present study demonstrated that CR accelerated cardiac autophagic flux in WT mice, but not in AMPKα2 KO mice, suggesting that the AMPK signaling pathway plays a crucial role in regulating cardiac autophagy in response to restricted caloric intake. It is conceivable that the AMPK-dependent autophagy not only provides a certain amount of energy but also promotes mitochondrial quality control to limit cardiac injury. Indeed, AMPK can simultaneously promote the destruction of defective mitochondria and the biogenesis of new mitochondria through PGC-1α, the net effect of which is the replenishment of new functional mitochondria and the attenuation of oxidative injury [76], [77].

Another interesting finding in the present study is that CR reduced heart weight in both wild type and AMPK KO mice, suggesting that AMPK signaling is not required for CR to reduce heart weight. This is in contrast with a previous study showing that loss of AMPKα2 exaggerated pressure overload-induced cardiac remodeling indicating AMPK as a negative regulator of cardiac hypertrophy [44]. However, there are multiple negative regulators of cardiac growth under diverse physiological and pathological conditions [78]. CR may induce heart size reduction through many pathways other than AMPK such as glycogen synthase kinase 3 (GSK-3) [79], liver X receptors [80]. thioredoxin 1 [81], and interferon regulatory factor 3 [82], to name only a few.

In summary, using AMPKα2 deficient mice, we demonstrated that in the absence of AMPK signaling, CR is unable to mount an effective hormetic response to protect the heart; instead, CR triggers adverse effects that culminate in cardiac dysfunction, suggesting that the AMPK signaling pathway is absolutely required for energy homeostasis and myocardial adaptation to restricted caloric intake. The results from this study may stimulate further research into the mechanisms of CR-induced cardioprotection, facilitating the design of effective mimetics to harness the power of CR for preventive and therapeutic intervention of heart disease.

## References

[1] Osborne TB, Mendel LB, Ferry EL (1917) The Effect of Retardation of Growth Upon the Breeding Period and Duration of Life of Rats. Science 45: 294–295. 17760202 10.1126/science.45.1160.294

[2] Martin B, Golden E, Egan JM, Mattson MP, Maudsley S (2007) Reduced energy intake: the secret to a long and healthy life? IBS J Sci 2: 35–39. 18985162 PMC2577199

[3] Masoro EJ (2009) Caloric restriction-induced life extension of rats and mice: A critique of proposed mechanisms. Biochim Biophys Acta. 10.1016/j.bbagen.2009.02.011 19250959

[4] Weindruch R (1996) The retardation of aging by caloric restriction: studies in rodents and primates. Toxicol Pathol 24: 742–745. 8994305 10.1177/019262339602400618

[5] Colman RJ, Anderson RM, Johnson SC, Kastman EK, Kosmatka KJ, et al (2009) Caloric restriction delays disease onset and mortality in rhesus monkeys. Science 325: 201–204. 19590001 10.1126/science.1173635 PMC2812811

[6] Mattison JA, Roth GS, Beasley TM, Tilmont EM, Handy AM, et al (2012) Impact of caloric restriction on health and survival in rhesus monkeys from the NIA study. Nature 489: 318–321. 22932268 10.1038/nature11432 PMC3832985

[7] Spindler SR (2010) Caloric restriction: from soup to nuts. Ageing Res Rev 9: 324–353. 19853062 10.1016/j.arr.2009.10.003

[8] Mattson MP, Wan R (2005) Beneficial effects of intermittent fasting and caloric restriction on the cardiovascular and cerebrovascular systems. J Nutr Biochem 16: 129–137. 15741046 10.1016/j.jnutbio.2004.12.007

[9] Cruzen C, Colman RJ (2009) Effects of caloric restriction on cardiovascular aging in non-human primates and humans. Clin Geriatr Med 25: 733–743, ix-x. 10.1016/j.cger.2009.07.001 PMC2786902 19944270

[10] Fontana L, Meyer TE, Klein S, Holloszy JO (2004) Long-term calorie restriction is highly effective in reducing the risk for atherosclerosis in humans. Proc Natl Acad Sci U S A 101: 6659–6663. 15096581 10.1073/pnas.0308291101 PMC404101

[11] Raeini-Sarjaz M, Vanstone CA, Papamandjaris AA, Wykes LJ, Jones PJ (2001) Comparison of the effect of dietary fat restriction with that of energy restriction on human lipid metabolism. Am J Clin Nutr 73: 262–267. 11157322 10.1093/ajcn/73.2.262

[12] Muthukumar A, Zaman K, Lawrence R, Barnes JL, Fernandes G (2003) Food restriction and fish oil suppress atherogenic risk factors in lupus-prone (NZB x NZW) F1 mice. J Clin Immunol 23: 23–33. 12645857 10.1023/a:1021996130672

[13] Viljanen AP, Karmi A, Borra R, Parkka JP, Lepomaki V, et al (2009) Effect of caloric restriction on myocardial fatty acid uptake, left ventricular mass, and cardiac work in obese adults. Am J Cardiol 103: 1721–1726. 19539082 10.1016/j.amjcard.2009.02.025

[14] Meyer TE, Kovacs SJ, Ehsani AA, Klein S, Holloszy JO, et al (2006) Long-term caloric restriction ameliorates the decline in diastolic function in humans. J Am Coll Cardiol 47: 398–402. 16412867 10.1016/j.jacc.2005.08.069

[15] Riordan MM, Weiss EP, Meyer TE, Ehsani AA, Racette SB, et al (2008) The effects of caloric restriction- and exercise-induced weight loss on left ventricular diastolic function. Am J Physiol Heart Circ Physiol 294: H1174–1182. 18162561 10.1152/ajpheart.01236.2007

[16] Pamplona R, Portero-Otin M, Requena J, Gredilla R, Barja G (2002) Oxidative, glycoxidative and lipoxidative damage to rat heart mitochondrial proteins is lower after 4 months of caloric restriction than in age-matched controls. Mech Ageing Dev 123: 1437–1446. 12425950 10.1016/s0047-6374(02)00076-3

[17] Williams TD, Chambers JB, Henderson RP, Rashotte ME, Overton JM (2002) Cardiovascular responses to caloric restriction and thermoneutrality in C57BL/6J mice. Am J Physiol Regul Integr Comp Physiol 282: R1459–1467. 11959690 10.1152/ajpregu.00612.2001

[18] Dhahbi JM, Tsuchiya T, Kim HJ, Mote PL, Spindler SR (2006) Gene expression and physiologic responses of the heart to the initiation and withdrawal of caloric restriction. J Gerontol A Biol Sci Med Sci 61: 218–231. 16567370 10.1093/gerona/61.3.218

[19] Dolinsky VW, Morton JS, Oka T, Robillard-Frayne I, Bagdan M, et al (2010) Calorie restriction prevents hypertension and cardiac hypertrophy in the spontaneously hypertensive rat. Hypertension 56: 412–421. 20696994 10.1161/HYPERTENSIONAHA.110.154732

[20] Shinmura K, Tamaki K, Bolli R (2008) Impact of 6-mo caloric restriction on myocardial ischemic tolerance: possible involvement of nitric oxide-dependent increase in nuclear Sirt1. Am J Physiol Heart Circ Physiol 295: H2348–2355. 18931029 10.1152/ajpheart.00602.2008 PMC2614541

[21] Shinmura K, Tamaki K, Saito K, Nakano Y, Tobe T, et al (2007) Cardioprotective effects of short-term caloric restriction are mediated by adiponectin via activation of AMP-activated protein kinase. Circulation 116: 2809–2817. 18040027 10.1161/CIRCULATIONAHA.107.725697

[22] Wan R, Ahmet I, Brown M, Cheng A, Kamimura N, et al.. (2009) Cardioprotective effect of intermittent fasting is associated with an elevation of adiponectin levels in rats. J Nutr Biochem. 10.1016/j.jnutbio.2009.01.020 PMC2854256 19423320

[23] Katare RG, Kakinuma Y, Arikawa M, Yamasaki F, Sato T (2009) Chronic intermittent fasting improves the survival following large myocardial ischemia by activation of BDNF/VEGF/PI3K signaling pathway. J Mol Cell Cardiol 46: 405–412. 19059263 10.1016/j.yjmcc.2008.10.027

[24] Ingram DK, Zhu M, Mamczarz J, Zou S, Lane MA, et al (2006) Calorie restriction mimetics: an emerging research field. Aging Cell 5: 97–108. 16626389 10.1111/j.1474-9726.2006.00202.x

[25] Lane MA, Roth GS, Ingram DK (2007) Caloric restriction mimetics: a novel approach for biogerontology. Methods Mol Biol 371: 143–149. 17634579 10.1007/978-1-59745-361-5_11

[26] Rattan SI (2004) Aging, anti-aging, and hormesis. Mech Ageing Dev 125: 285–289. 15063104 10.1016/j.mad.2004.01.006

[27] Sohal RS, Weindruch R (1996) Oxidative stress, caloric restriction, and aging. Science 273: 59–63. 8658196 10.1126/science.273.5271.59 PMC2987625

[28] Chung HY, Kim HJ, Kim JW, Yu BP (2001) The inflammation hypothesis of aging: molecular modulation by calorie restriction. Ann N Y Acad Sci 928: 327–335. 11795524

[29] Cohen HY, Miller C, Bitterman KJ, Wall NR, Hekking B, et al (2004) Calorie restriction promotes mammalian cell survival by inducing the SIRT1 deacetylase. Science 305: 390–392. 15205477 10.1126/science.1099196

[30] Spindler SR (2009) Caloric restriction: From soup to nuts. Ageing Res Rev. 10.1016/j.arr.2009.10.003 19853062

[31] Schumacher B, van der Pluijm I, Moorhouse MJ, Kosteas T, Robinson AR, et al (2008) Delayed and accelerated aging share common longevity assurance mechanisms. PLoS Genet 4: e1000161. 18704162 10.1371/journal.pgen.1000161 PMC2493043

[32] Viollet B, Athea Y, Mounier R, Guigas B, Zarrinpashneh E, et al (2009) AMPK: Lessons from transgenic and knockout animals. Front Biosci 14: 19–44. 10.2741/3229 PMC2666987 19273052

[33] McCarty MF (2004) Chronic activation of AMP-activated kinase as a strategy for slowing aging. Med Hypotheses 63: 334–339. 15236799 10.1016/j.mehy.2004.01.043

[34] Greer EL, Banko MR, Brunet A (2009) AMP-activated protein kinase and FoxO transcription factors in dietary restriction-induced longevity. Ann N Y Acad Sci 1170: 688–692. 19686213 10.1111/j.1749-6632.2009.04019.x PMC2814416

[35] Narbonne P, Roy R (2009) Caenorhabditis elegans dauers need LKB1/AMPK to ration lipid reserves and ensure long-term survival. Nature 457: 210–214. 19052547 10.1038/nature07536

[36] Canto C, Auwerx J (2011) Calorie restriction: is AMPK a key sensor and effector? Physiology (Bethesda) 26: 214–224. 21841070 10.1152/physiol.00010.2011 PMC3627048

[37] Viollet B, Andreelli F, Jorgensen SB, Perrin C, Geloen A, et al (2003) The AMP-activated protein kinase alpha2 catalytic subunit controls whole-body insulin sensitivity. J Clin Invest 111: 91–98. 12511592 10.1172/JCI16567 PMC151837

[38] Tang YD, Kuzman JA, Said S, Anderson BE, Wang X, et al (2005) Low thyroid function leads to cardiac atrophy with chamber dilatation, impaired myocardial blood flow, loss of arterioles, and severe systolic dysfunction. Circulation 112: 3122–3130. 16275864 10.1161/CIRCULATIONAHA.105.572883

[39] Chen J, Shearer GC, Chen Q, Healy CL, Beyer AJ, et al (2011) Omega-3 fatty acids prevent pressure overload-induced cardiac fibrosis through activation of cyclic GMP/protein kinase G signaling in cardiac fibroblasts. Circulation 123: 584–593. 21282499 10.1161/CIRCULATIONAHA.110.971853 PMC3056077

[40] Kobayashi S, Mao K, Zheng H, Wang X, Patterson C, et al (2007) Diminished GATA4 protein levels contribute to hyperglycemia-induced cardiomyocyte injury. J Biol Chem 282: 21945–21952. 17525155 10.1074/jbc.M703048200

[41] Chen K, Xu X, Kobayashi S, Timm D, Jepperson T, et al (2011) Caloric restriction mimetic 2-deoxyglucose antagonizes doxorubicin-induced cardiomyocyte death by multiple mechanisms. J Biol Chem 286: 21993–22006. 21521688 10.1074/jbc.M111.225805 PMC3121344

[42] Shioji K, Kishimoto C, Nakamura H, Masutani H, Yuan Z, et al (2002) Overexpression of thioredoxin-1 in transgenic mice attenuates adriamycin-induced cardiotoxicity. Circulation 106: 1403–1409. 12221060 10.1161/01.cir.0000027817.55925.b4

[43] Edwards AG, Donato AJ, Lesniewski LA, Gioscia RA, Seals DR, et al (2010) Life-long caloric restriction elicits pronounced protection of the aged myocardium: a role for AMPK. Mech Ageing Dev 131: 739–742. 20934448 10.1016/j.mad.2010.09.007 PMC3010207

[44] Zhang P, Hu X, Xu X, Fassett J, Zhu G, et al (2008) AMP activated protein kinase-alpha2 deficiency exacerbates pressure-overload-induced left ventricular hypertrophy and dysfunction in mice. Hypertension 52: 918–924. 18838626 10.1161/HYPERTENSIONAHA.108.114702 PMC2760829

[45] Hepple RT (2009) Why eating less keeps mitochondria working in aged skeletal muscle. Exerc Sport Sci Rev 37: 23–28. 19098521 10.1097/JES.0b013e3181877dc5

[46] Appenzeller-Herzog C, Hall MN (2012) Bidirectional crosstalk between endoplasmic reticulum stress and mTOR signaling. Trends Cell Biol 22: 274–282. 22444729 10.1016/j.tcb.2012.02.006

[47] Wohlgemuth SE, Julian D, Akin DE, Fried J, Toscano K, et al (2007) Autophagy in the heart and liver during normal aging and calorie restriction. Rejuvenation Res 10: 281–292. 17665967 10.1089/rej.2006.0535

[48] Kuma A, Hatano M, Matsui M, Yamamoto A, Nakaya H, et al (2004) The role of autophagy during the early neonatal starvation period. Nature 432: 1032–1036. 15525940 10.1038/nature03029

[49] Matsui Y, Takagi H, Qu X, Abdellatif M, Sakoda H, et al (2007) Distinct roles of autophagy in the heart during ischemia and reperfusion: roles of AMP-activated protein kinase and Beclin 1 in mediating autophagy. Circ Res 100: 914–922. 17332429 10.1161/01.RES.0000261924.76669.36

[50] Nagata D, Hirata Y (2009) The role of AMP-activated protein kinase in the cardiovascular system. Hypertens Res 33: 22–28. 19911004 10.1038/hr.2009.187

[51] Hoyer-Hansen M, Jaattela M (2007) AMP-activated protein kinase: a universal regulator of autophagy? Autophagy 3: 381–383. 17457036 10.4161/auto.4240

[52] Nakai A, Yamaguchi O, Takeda T, Higuchi Y, Hikoso S, et al (2007) The role of autophagy in cardiomyocytes in the basal state and in response to hemodynamic stress. Nat Med 13: 619–624. 17450150 10.1038/nm1574

[53] Mizushima N, Yoshimori T (2007) How to Interpret LC3 Immunoblotting. Autophagy 3. 10.4161/auto.4600 17611390

[54] Pankiv S, Clausen TH, Lamark T, Brech A, Bruun JA, et al (2007) p62/SQSTM1 binds directly to Atg8/LC3 to facilitate degradation of ubiquitinated protein aggregates by autophagy. J Biol Chem 282: 24131–24145. 17580304 10.1074/jbc.M702824200

[55] Komatsu M, Waguri S, Koike M, Sou YS, Ueno T, et al (2007) Homeostatic Levels of p62 Control Cytoplasmic Inclusion Body Formation in Autophagy-Deficient Mice. Cell 131: 1149–1163. 18083104 10.1016/j.cell.2007.10.035

[56] Mizushima N, Yoshimori T (2007) How to interpret LC3 immunoblotting. Autophagy 3: 542–545. 17611390 10.4161/auto.4600

[57] Jung CH, Jun CB, Ro SH, Kim YM, Otto NM, et al (2009) ULK-Atg13-FIP200 complexes mediate mTOR signaling to the autophagy machinery. Mol Biol Cell 20: 1992–2003. 19225151 10.1091/mbc.E08-12-1249 PMC2663920

[58] Mizushima N (2010) The role of the Atg1/ULK1 complex in autophagy regulation. Curr Opin Cell Biol 22: 132–139. 20056399 10.1016/j.ceb.2009.12.004

[59] Kim J, Kundu M, Viollet B, Guan KL (2011) AMPK and mTOR regulate autophagy through direct phosphorylation of Ulk1. Nat Cell Biol 13: 132–141. 21258367 10.1038/ncb2152 PMC3987946

[60] Bach M, Larance M, James DE, Ramm G (2011) The serine/threonine kinase ULK1 is a target of multiple phosphorylation events. Biochem J 440: 283–291. 21819378 10.1042/BJ20101894

[61] Egan DF, Shackelford DB, Mihaylova MM, Gelino S, Kohnz RA, et al (2011) Phosphorylation of ULK1 (hATG1) by AMP-activated protein kinase connects energy sensing to mitophagy. Science 331: 456–461. 21205641 10.1126/science.1196371 PMC3030664

[62] Beauloye C, Bertrand L, Horman S, Hue L (2011) AMPK, a potential therapeutic target against the transition from cardiac injury to heart failure. Cardiovasc Res. 10.1093/cvr/cvr034 21285292

[63] Kim M, Tian R (2011) Targeting AMPK for cardiac protection: Opportunities and challenges. J Mol Cell Cardiol. 10.1016/j.yjmcc.2010.12.004 PMC3078514 21147121

[64] Maslov MY, Chacko VP, Stuber M, Moens AL, Kass DA, et al (2007) Altered high-energy phosphate metabolism predicts contractile dysfunction and subsequent ventricular remodeling in pressure-overload hypertrophy mice. Am J Physiol Heart Circ Physiol 292: H387–391. 16963614 10.1152/ajpheart.00737.2006

[65] Salminen A, Kaarniranta K (2012) AMP-activated protein kinase (AMPK) controls the aging process via an integrated signaling network. Ageing Res Rev 11: 230–241. 22186033 10.1016/j.arr.2011.12.005

[66] Crow MT, Mani K, Nam YJ, Kitsis RN (2004) The mitochondrial death pathway and cardiac myocyte apoptosis. Circ Res 95: 957–970. 15539639 10.1161/01.RES.0000148632.35500.d9

[67] Ferri KF, Kroemer G (2001) Organelle-specific initiation of cell death pathways. Nat Cell Biol 3: E255–263. 11715037 10.1038/ncb1101-e255

[68] Terai K, Hiramoto Y, Masaki M, Sugiyama S, Kuroda T, et al (2005) AMP-activated protein kinase protects cardiomyocytes against hypoxic injury through attenuation of endoplasmic reticulum stress. Mol Cell Biol 25: 9554–9575. 16227605 10.1128/MCB.25.21.9554-9575.2005 PMC1265833

[69] Shibata R, Sato K, Pimentel DR, Takemura Y, Kihara S, et al (2005) Adiponectin protects against myocardial ischemia-reperfusion injury through AMPK- and COX-2-dependent mechanisms. Nat Med 11: 1096–1103. 16155579 10.1038/nm1295 PMC2828682

[70] Russell RR III, Li J, Coven DL, Pypaert M, Zechner C, et al (2004) AMP-activated protein kinase mediates ischemic glucose uptake and prevents postischemic cardiac dysfunction, apoptosis, and injury. J Clin Invest 114: 495–503. 15314686 10.1172/JCI19297 PMC503766

[71] Gottlieb RA, Carreira RS (2010) Autophagy in health and disease. 5. Mitophagy as a way of life. Am J Physiol Cell Physiol 299: C203–210. 20357180 10.1152/ajpcell.00097.2010 PMC2928637

[72] Gottlieb RA, Gustafsson AB (2010) Mitochondrial turnover in the heart. Biochim Biophys Acta 1813: 1295–1301. 21147177 10.1016/j.bbamcr.2010.11.017 PMC3335292

[73] Bergamini E, Cavallini G, Donati A, Gori Z (2007) The role of autophagy in aging: its essential part in the anti-aging mechanism of caloric restriction. Ann N Y Acad Sci 1114: 69–78. 17934054 10.1196/annals.1396.020

[74] Madeo F, Tavernarakis N, Kroemer G (2010) Can autophagy promote longevity? Nat Cell Biol 12: 842–846. 20811357 10.1038/ncb0910-842

[75] Jia K, Levine B (2007) Autophagy is required for dietary restriction-mediated life span extension in C. elegans. Autophagy 3: 597–599. 17912023 10.4161/auto.4989

[76] Mihaylova MM, Shaw RJ (2011) The AMPK signalling pathway coordinates cell growth, autophagy and metabolism. Nat Cell Biol 13: 1016–1023. 21892142 10.1038/ncb2329 PMC3249400

[77] Jornayvaz FR, Shulman GI (2010) Regulation of mitochondrial biogenesis. Essays Biochem 47: 69–84. 20533901 10.1042/bse0470069 PMC3883043

[78] Hardt SE, Sadoshima J (2004) Negative regulators of cardiac hypertrophy. Cardiovasc Res 63: 500–509. 15276475 10.1016/j.cardiores.2004.03.015

[79] Cheng H, Woodgett J, Maamari M, Force T (2011) Targeting GSK-3 family members in the heart: a very sharp double-edged sword. J Mol Cell Cardiol 51: 607–613. 21163265 10.1016/j.yjmcc.2010.11.020 PMC3075376

[80] Wu S, Yin R, Ernest R, Li Y, Zhelyabovska O, et al (2009) Liver X receptors are negative regulators of cardiac hypertrophy via suppressing NF-kappaB signalling. Cardiovasc Res 84: 119–126. 19487338 10.1093/cvr/cvp180 PMC2741346

[81] Yang Y, Ago T, Zhai P, Abdellatif M, Sadoshima J (2011) Thioredoxin 1 negatively regulates angiotensin II-induced cardiac hypertrophy through upregulation of miR-98/let-7. Circ Res 108: 305–313. 21183740 10.1161/CIRCRESAHA.110.228437 PMC3249645

[82] Lu J, Bian ZY, Zhang R, Zhang Y, Liu C, et al (2013) Interferon regulatory factor 3 is a negative regulator of pathological cardiac hypertrophy. Basic Res Cardiol 108: 326. 23307144 10.1007/s00395-012-0326-9

